# Is the Scottish population living dangerously? Prevalence of multiple risk factors: the Scottish Health Survey 2003

**DOI:** 10.1186/1471-2458-10-330

**Published:** 2010-06-11

**Authors:** Richard Lawder, Oliver Harding, Diane Stockton, Colin Fischbacher, David H Brewster, Jim Chalmers, Alan Finlayson, David I Conway

**Affiliations:** 1 Information Services Division, NHS National Services Scotland, Gyle Square, Edinburgh, UK; 2 Department of Public Health, NHS Forth Valley Health Board, Stirling, UK; 3 Department of Public Health Sciences, University of Edinburgh, Edinburgh, UK; 4 Dental School, Faculty of Medicine, University of Glasgow, Glasgow, UK

## Abstract

**Background:**

Risk factors are often considered individually, we aimed to investigate the prevalence of combinations of multiple behavioural risk factors and their association with socioeconomic determinants.

**Methods:**

Multinomial logistic regression was used to model the associations between socioeconomic factors and multiple risk factors from data in the Scottish Health Survey 2003. Prevalence of five key risk - smoking, alcohol, diet, overweight/obesity, and physical inactivity, and their risk in relation to demographic, individual and area socioeconomic factors were assessed.

**Results:**

Full data were available on 6,574 subjects (80.7% of the survey sample). Nearly the whole adult population (97.5%) reported to have at least one behavioural risk factor; while 55% have three or more risk factors; and nearly 20% have four or all five risk factors. The most important determinants for having four or five multiple risk factors were low educational attainment which conferred over a 3-fold increased risk compared to high education; and residence in the most deprived communities (relative to least deprived) which had greater than 3-fold increased risk.

**Conclusions:**

The prevalence of multiple behavioural risk factors was high and the prevalence of absence of all risk factors very low. These behavioural patterns were strongly associated with poorer socioeconomic circumstances. Policy to address factors needs to be joined up and better consider underlying socioeconomic circumstances.

## Background

The World Health Organisation's Global Burden of Diseases Project identified five risk factors which contribute around 90% of the total burden of disease in high income country populations: tobacco use, alcohol consumption, poor diet, physical inactivity, overweight and obesity [1]. While epidemiological evidence is usually gathered on a single-risk factor basis [2,3], risk factors occur in individuals and populations in different combinations, and may show additive or multiplicative interactions [4,5]. This has implications for interventions - is it better to focus on one risk factor at a time, or to encourage motivated individuals to make more wholesale changes in their lifestyle to address more than one risk factor at a time?

There are few population-based studies investigating the prevalence of combinations of risk factors [5-9]. Most studies focus on smoking; and while there is abundant evidence of the association with lifestyle and socioeconomic status, there is limited consideration of the relationship between combinations of multiple behaviours and socioeconomic factors.

Thus far analysis of risk factors in Scotland has been limited to individual risk factors such as smoking [2], alcohol [10] or diet [11]. Here we aim to use population data from the Scottish Health Survey to assess the prevalence of different combinations of multiple behavioural risk factors and to examine how these combined behaviours relate to area-based and individual socioeconomic factors.

## Methods

The 2003 Scottish Health Survey is a cross-sectional national population-based survey and is the third of a series of surveys, the first two of which took place in 1995 and 1998. Their aim is to monitor health status and health-related lifestyles in the Scottish population. Sampling was via a multi-stage stratified probability sampling design using postcode sectors selected at the first stage and household addresses at the second stage. The survey used weights to correct for survey design (large households were underrepresented) and non-response biases. The survey methodology is described in detail elsewhere [12] and will only briefly be described here. Face-to-face interviews took place in the subject's home using Computer Assisted Personal Interviewing (CAPI), and permission was sought for a follow-up visit from a specially trained nurse. The interview covered a range of items including: self assessed health and disability, health service use, cardiovascular and respiratory disease, smoking, drinking, common mental health problems, eating patterns and physical activity and information on a range of indicators of socioeconomic position. The nurse asked further questions, for example on use of prescribed medicines, made anthropometric and biomedical measurements, including blood pressure, waist and hip circumference and lung function and collected blood and saliva samples. Saliva samples were analysed for cotinine to validate self-reported smoking [12]. Original ethical approval for the Scottish Health Survery 2003 was granted by Multicentre Research Ethics Committees. Anonymised data are accessible via the UK Data Archive for which no additional ethical approval was required.

### Risk factor variables

Our analysis considered four lifestyle risk factors and obesity. Each factor was categorised in binary form - respondents either having or not having the risk factor. Smoking (including cigarettes, cigars, or pipe) was defined into two categories as: current smokers versus, collectively, those who never smoked regularly, never smoked at all (with "regularly" defined as once per day for a month), or ex-smokers. Data were validated by salivary cotinine analysis. Heavy alcohol consumption was defined as exceeding the UK Royal College of Physicians definition of sensible drinking (21 units/week for men and 14 units/week for women; 1 unit of alcohol is defined as 10 ml (8 grams) of ethanol) [13]. The dietary variable was defined by the WHO and national recommendation to consume five portions or more of fruit and vegetables daily [14,15]. Respondents were classified either as "not reaching the recommended daily intake", or "reaching the recommended daily intake". Overweight and obesity were classified following the 1999 definition of the International Obesity Task Force [16]. Thus, respondents having a BMI ≥ 25 kg/m^2 ^were classified as "overweight/obese" and those < 25 defined as "underweight/desirable". Questions on physical activity included number of days and minutes per day of participation in: heavy housework, heavy "Do-It-Yourself" (DIY)/gardening/home maintenance, walking for any purpose, and recreational sports and exercises. Being physically active was defined by participation in at least 30 minutes of moderate exercise on five or more days of the week - based on the Allied Dunbar National Fitness Survey criteria [17]. Respondents were classified as "Meeting the recommended level of physical activity - Physically Active"; otherwise they were classified as "Physically Inactive".

The following demographic and socioeconomic variables were also included in the analysis: sex (male, female); age (grouped in the following categories: 16-39, 40-64, 65+ years); highest educational qualification (degree level or above, below degree level, no qualifications); ethnicity (white versus black and minority ethnic (BME) groups); marital status (never married, currently married, divorced/separated/widowed); economic activity status (employed, unemployed, retired, economically inactive) and the Registrar General's Occupational Social Class (I, II, III VI, V, other) for the household chief income earner. The Scottish Index of Multiple Deprivation (SIMD 2006), an area-based level of deprivation, was derived from the residential postcode of the respondents and categorised into quintiles - 1 (least deprived) to 5 (most deprived) [18]. The SIMD score is calculated at the level of "data zones" using 37 indicators from a range of administrative data sources grouped into seven domains: income, employment, housing, health, education, geographical access to services/telecommunications and crime. Data zones are stable and consistent small geographical areas in Scotland, grouped from 2001 Census Output Areas, and have populations of between 500 and 1,000 residents nested within Local Authority boundaries. They are intended to be effective at identifying small areas with similar social and economic characteristics [18].

### Statistical analysis

Risk factor prevalence and 95% confidence intervals (CI) were calculated. To ensure accurately computed estimates of the population statistics and their standard errors, sample design characteristics including stratification, multi-stage cluster sampling and probability sampling weights were taken into account. Cross-tabulations were performed to show all possible clustering patterns of the five risk factors presented.

Relative Risk Ratios (RRR) and 95% confidence intervals were computed using multinomial logistic regression modelling to examine the independent association between each covariate and the dependent ordinal risk factor variable taking on the following four levels (zero or one risk factor, two risk factors, three risk factors, and four or five risk factors), comparing to the reference group of zero or one risk factor. The model included age, sex, ethnicity, education, marital status, economic activity status, occupational social class, and area-based socioeconomic circumstance (SIMD 2006). All statistical analyses were performed using Stata version 8.0 (Stata Corporation, College Station, TX, USA).

## Results

The 2003 Scottish Health Survey included 8,148 adult respondents - 4,538 females and 3,610 males - representing a 67% response rate for eligible households. Full data on all five risk factors were available for 80.7% (n = 6,574) of the sample, representing 54% overall response rate. Tablez 1 presents the demographic and socioeconomic profile and the prevalence of risk factors. There were more women than men in the sample, women were slightly older than men, and there were some marked differences between SIMD quintiles - particularly a lower response in those from relatively more deprived areas. Only around 2% of the sample were from BME groups and a half of men and women were currently married. Just over 20% of men and women were educated to the highest level but more women had no qualifications. There were substantially more men than women currently employed - although for those in employment the occupational social class distribution was similar. Fruit and vegetable consumption was similar in both sexes, while smoking, excessive alcohol consumption and overweight/obesity were more common and physical inactivity less common amongst men (Table 1).

**Table 1:** Demographic, socioeconomic and behavioural risk factor profile of study sample by sex

	Men (n = 2,941) % (95%CI)	Women (n = 3,633) % (95%CI)	Total (n = 6,574) % (95%CI)
**Age (years)**			
16-24	14.7 (12.6, 17.0)	12.4 (10.9, 14.1)	13.5 (12.1, 15.0)
25-34	16.0 (14.3, 17.7)	15.7 (14.4, 17.1)	15.8 (14.6, 17.1)
35-44	19.9 (18.4, 21.6)	20.1 (18.7, 21.7)	20.0 (18.8, 21.4)
45-54	17.7 (16.2, 19.4)	16.9 (15.7, 18.1)	17.3 (16.2, 18.4)
55-64	15.3 (13.9, 16.9)	15.0 (13.8, 16.1)	15.1 (14.1, 16.3)
65 and over	16.4 (15.0, 17.8)	20.0 (18.5, 21.5)	18.2 (17.0, 19.5)

**Deprivation (SIMD2006 quintile)**			
1 (least deprived)	22.1 (19.7-24.7)	21.3 (19.1-23.6)	21.7 (19.5-24.0)
2	21.2 (18.9-23.7)	20.2 (18.1-22.5)	20.7 (18.7-22.9)
3	20.2 (17.7-22.9)	19.9 (17.7-22.3)	20.0 (17.8-22.5)
4	19.6 (17.3-22.1)	19.6 (17.7-21.7)	19.6 (17.7-21.7)
5 (most deprived)	17.0 (15.3-18.8)	19.0 (17.3-20.9)	18.0 (16.5-19.7)

**Education**			
Degree level or above	24.0 (22.0-26.1)	22.7 (20.8-24.7)	23.3 (21.64-25.12)
Below degree level	45.6 (43.4-47.7)	41.2 (39.0-43.4)	43.3 (41.63-44.93)
No qualifications	30.4 (28.4-32.5)	36.0 (34.2-37.9)	33.3 (31.75-34.92)
Missing	0.1 (0.0-0.3)	0.1(0.0-0.3)	0.1 (0.0-0.2)

**Ethnicity**			
White	96.8 (95.7-97.7)	97.9 (97.1-98.4)	97.4 (96.6-97.4)
Black and Minority Ethnic group	2.7 (1.9-3.8)	1.8 (1.3-2.5)	2.2 (1.7-3.0)
Missing	0.5 (0.2-0.9)	0.4 (0.2-0.7)	0.4 (0.3-0.7)

**Marital status**			
Never married	31.9 (29.7-34.1)	25.2 (23.2-27.3)	28.4 (26.7-30.2)
Currently married	56.2 (54.0-58.4)	51.1 (49.0-53.2)	53.6 (51.7-55.5)
Divorced, separated or widowed	11.8 (10.8-12.9)	23.6 (22.2-25.1)	17.9 (17.0-19.0)
Missing	0.1 (0.0-0.3)	0.1 (0.0-0.2)	0.1 (0.0-0.3)

**Economic activity status**			
Employed	62.8 (60.7-64.9)	49.9 (48.0-51.8)	56.1 (54.5-57.7)
Unemployed	6.5 (5.5-7.8)	5.0 (4.1-6.0)	5.7 (5.0-6.6)
Retired	17.8 (16.3-19.4)	21.8 (20.3-23.3)	19.9 (18.6-21.2)
Economically inactive	12.7 (11.3-14.3)	23.1 (21.6-24.7)	18.1 (17.0-19.3)
Missing	0.1 (0.0-0.5)	0.2 (0.1-0.7)	0.2 (0.1-0.4)

**Occupational social class**			
I - professional	5.3 (4.3-6.5)	5.0 (4.2-6.1)	5.1 (4.4-6.0)
II - managerial and technical	20.7 (18.9-22.7)	20.3 (18.9-21.9)	20.5 (19.2-21.9)
III - skilled	48.3 (46.2-50.5)	48.1 (46.1-50.1)	48.2 (46.5-49.9)
IV - partly-skilled	15.6 (14.2-17.1)	15.4 (14.0-16.9)	15.5 (14.4-16.6)
V - unskilled	6.4 (5.5-7.5)	8.0 (7.1-9.1)	7.3 (6.5-8.1)
Others	1.1 (0.8-1.6)	1.1 (0.8-1.5)	1.1 (0.9-1.4)
Missing	2.6 (1.9-3.6)	2.1 (1.6-2.8)	2.4 (1.9-3.0)

**Smoking status**			
Current	33.8 (31.8-35.8)	30.3 (28.6-32.1)	32.0 (30.6, 33.4)

**Drinking status**			
> recommended sensible level*	28.2 (26.4-30.0)	15.2 (13.8-16.7)	21.4 (20.2, 22.6)

**Fruit and vegetable consumption**			
< 5 portions/day	79.6 (77.8-81.2)	77.1 (75.4-78.6)	78.3 (76.9, 79.6)

**Overweight/obesity**			
BMI ≥ 25 kg/m^2^	65.8 (64.2-67.5)	60.0 (58.2-61.8)	62.8 (61.4, 64.1)

**Physical activity**			
< 5 episodes/week	59.2 (57.1-61.4)	70.5 (68.8-72.1)	65.1 (63.6, 66.6)

* > 21 units/week for men, > 14 units/week women

CI - Confidence Interval

BMI - Body Mass Index

SIMD - Scottish Index of Multiple Deprivation (2006)

### Multiple risk factors

**Figure 1:**
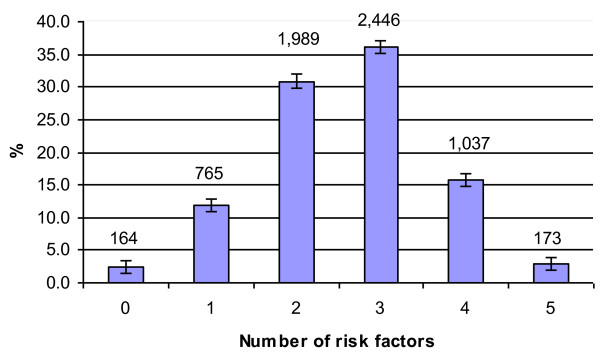
**Risk factor prevalence and 95% Confidence Intervals among the adult population**. numbers indicated above bars represent respondents

Figure 1 presents the prevalence of the combined multiple risk factors. The following summarises different combinations of risk factors:

•*Number of risk factors *- Only 2.5% of the sample had no risk factors. 88.2% had more than one risk factor.

•*Risk factors in non-smokers drinking alcohol within recommended limits *- Nearly 20% of the population surveyed were overweight/obese, physically inactive and had a poor diet without other risk factors (Table 2); 10% were physically inactive and had a poor diet; 8% were physically active but had a poor diet and were overweight/obese.

**Table 2:** Ranked prevalence of all risk factors and combinations of multiple behavioural risk factors

	Risk factor					
**Number of risk factors**	**Smoking**	**Risky alcohol drinking**	**BMI ≥ 25 kg/m^2^**	**Physically inactive**	**Diet low in fruit and veg**	**Prevalence (%)**

5	Y	Y	Y	Y	Y	2.9 (2.4,2.5)



4	Y	N	Y	Y	Y	7.7 (7.0,8.5)

4	N	Y	Y	Y	Y	4.0 (3.5,4.6)

4	Y	Y	N	Y	Y	2.0 (1.7,2.4)

4	Y	Y	Y	N	Y	1.6 (1.3,2.0)

4	Y	Y	Y	Y	N	0.5 (0.3,0.5)



3	N	N	Y	Y	Y	19.5 (18.5,20.6)

3	Y	N	N	Y	Y	5.9 (5.3,6.5)

3	Y	N	Y	N	Y	3.1 (2.7,3.6)

3	N	Y	Y	N	Y	2.4 (2.0,2.8)

3	Y	Y	N	N	Y	1.5 (1.2,1.9)

3	N	Y	N	Y	Y	1.4 (1.1,1.7)

3	Y	N	Y	Y	N	1.0 (0.8,1.4)

3	N	Y	Y	Y	N	0.9 (0.7,1.3)

3	Y	Y	Y	N	N	0.3 (0.2,0.5)

3	Y	Y	N	Y	N	0.1 (0.1,0.3)



2	N	N	N	Y	Y	9.7 (8.8,10.7)

2	N	N	Y	N	Y	8.0 (7.3,8.7)

2	N	N	Y	Y	N	5.8 (5.2,6.4)

2	Y	N	N	N	Y	2.9 (2.4,3.5)

2	N	Y	N	N	Y	1.3 (0.9,1.7)

2	N	Y	Y	N	N	1.1 (0.8,1.4)

2	Y	N	N	Y	N	0.7 (0.5,1.0)

2	Y	N	Y	N	N	0.6 (0.4,0.9)

2	N	Y	N	Y	N	0.5 (0.3,0.7)

2	Y	Y	N	N	N	0.4 (0.3,0.6)



1	N	N	N	N	Y	4.6 (4.0,5.1)

1	N	N	Y	N	N	3.4 (2.9,3.9)

1	N	N	N	Y	N	2.5 (2.1,3.0)

1	Y	N	N	N	N	0.7 (0.5,1.0)

1	N	Y	N	N	N	0.6 (0.4,1.0)

Y = included in combination of risk factors

N = excluded in combination of risk factors

BMI - Body Mass Index

•*Risk factors in smokers and risk alcohol drinkers *-The combination of excessive alcohol consumption and smoking was found in 9% of respondents. This group tended to have more additional risk factors - nearly three quarters had a total of 4 or 5 risk factors present, the majority being overweight or obese. Of those who drank to excess but did not smoke (12%), 70% were overweight or obese, usually in combination with poor diet, physical inactivity or both. Of those who smoked but did not drink to excess (23%) more than half were overweight or obese (97% of overweight/obese smokers having poor diet, taking insufficient physical activity or both).

Relative to zero or one risk factor, combinations of two or three multiple risk factors were significantly more common among men than women, and among those living in the most deprived communities relative to least deprived communities (Table 3). This association was also observed for those with relatively low educational attainment or lower occupational social class. Retired groups had significantly greater probability of multiple risk factors than other levels of economic activity. BME groups had lower probability of combinations of two or three risk factors compared to white counterparts. Similar but generally much stronger results were observed for combinations of four or five multiple risk factors but increased probabilities were also associated with those in economically inactive groups and in those with divorced, separated or widowed marital status.

**Table 3:** Multinomial logistic regression for combinations of multiple risk factors^1^, in adults aged 16 years and over. All factors are mutually adjusted for each other

Variable (n)	(2) vs (0 or 1) risk factors^1^	(3) vs (0 or 1) risk factors^1^	(4 or 5) vs (0 or 1) risk factors1
	**RRR (95% CI)**		

**Sex**			

^†^Women (3,663)	1.00	1.00	1.00

Men (2,941)	1.12 (0.93,1.33) n/s	1.28 (1.09,1.50) **	1.81 (1.51,2.19) ***

**Age**			

^†^16-24 (567)	1.00		1.00

25-34 (860)	1.08 (0.75,1.56) n/s	1.70 (1.10,2.62) *	1.65 (1.06,2.56) *

35-44 (1,357)	1.20 (0.80,1.80) n/s	1.84 (1.25,2.71) **	1.74 (1.15,2.63) **

45-54 (1,178)	1.06 (0.69,1.62) n/s	1.90 (1.22,2.95) **	1.79 (1.13,2.82) *

55-64 (1,203)	1.19 (0.78,1.81) n/s	1.84 (1.15,2.95) *	1.51 (0.92,2.48) n/s

65+ (1,409)	1.19 (0.66,2.17) n/s	2.00 (1.08,3.71) *	0.88 (0.48,1.64) n/s

**Deprivation (SIMD2006 quintile)**			

^†^1 (Least Deprived) (1,313)	1.00	1.00	1.00

2 (1,425)	0.96 (0.74,1.26) n/s	0.92 (0.71,1.2) n/s	0.98 (0.70,1.36) n/s

3 (1,467)	0.93 (0.69,1.24) n/s	1.01 (0.77,1.31) n/s	1.09 (0.78,1.52) n/s

4 (1,265)	1.04 (0.78,1.38) n/s	1.25 (0.95,1.65) n/s	1.35 (1.00,1.82) n/s

5 (Most Deprived) (1,104)	1.75 (1.24,2.48) **	2.21 (1.60,3.06) ***	3.20 (2.28,4.49) ***

**Highest Educational Qualification**			

^†^Degree or above (1,518)	1.00		1.00

Below degree level (2,626)	1.33 (1.06,1.67) *	1.68 (1.37,2.05) ***	1.90 (1.49,2.41) ***

No qualifications (2,426)	1.86 (1.40,2.48) ***	3.18 (2.38,4.25) ***	3.14 (2.31,4.26) ***

**Race/ethnicity**			

^†^White (6,440)	1.00		1.00

BME (109)	0.44 (0.23,0.83) *	0.32 (0.16,0.65) **	0.16 (0.06,0.41) ***

**Marital Status**			

^†^Never Married (1,496)	1.00	1.00	1.00

Currently Married (3,744)	0.86 (0.69,1.09) n/s	1.01 (0.79,1.30) n/s	1.04 (0.78,1.39) n/s

Divorced, separated, widowed (1,329)	1.01 (0.72,1.41) n/s	1.10 (0.77,1.56) n/s	1.46 (0.99,2.16) n/s

**Economic Activity Status**			

^†^Employed (3,540)	1.00	1.00	1.00

Unemployed (295)	0.90 (0.57,1.42) n/s	0.98 (0.65,1.45) n/s	0.99 (0.61,1.61) n/s

Retired (1,555)	1.63 (1.10,2.42) *	1.66 (1.16,2.37) **	2.07 (1.38,3.10) ***

Economically inactive (1,177)	0.98 (0.73,1.31) n/s	1.13 (0.88,1.47) n/s	1.60 (1.21,2.11) **

**Occupational Social Class**			

^†^I professional (325)	1.00	1.00	1.00

II managerial & technical (1,384)	1.16 (0.79,1.69) n/s	1.05 (0.74,1.49) n/s	1.11 (0.68,1.80) n/s

III skilled (3,132)	1.70 (1.18,2.46) **	1.42 (1.00,2.04) n/s	2.05 (1.24,3.39) **

IV partly skilled (1,055)	1.65 (1.10,2.48) *	1.46 (1.01,2.11) *	1.89 (1.15,3.12) *

V unskilled (490)	1.70 (1.05,2.76) *	1.72 (1.04,2.83) *	2.19 (1.17,4.10) *

Others (77)	1.50 (0.51,4.43) n/s	1.60 (0.61,4.22) n/s	1.78 (0.57,5.5) n/s

1. Risk factors are current smoker, risky alcohol drinking, BMI ≥ 25 kg/m2, physically inactive, and poor diet

RRR = relative risk ratios; CI = Confidence Interval; * = p < 0.05; ** = p < 0.01; *** = p < 0.001; n/s = not significant

†. Reference category; Note: N may not add up to sample size (6,574) for some variables due to unanswered questions

Of all factors assessed those living in the most deprived areas and those with no educational qualifications had the greatest probability of accumulating multiple behavioural risk factors, with over a 3 fold increase associated with combinations of four or five risk factors. The SIMD profile of those included with all five variables (80%) and those excluded in the analysis (20%) were significantly correlated, p = 0.02 (data not shown).

## Discussion

The Scottish population seems to be living dangerously. Considering five major risk factors to health - cigarette smoking, heavy alcohol consumption, poor diet, physical inactivity, and overweight - nearly the whole adult population (97.5%) have at least one behavioural risk factor; 86% have at least two risk factors; 55% have three or more risk factors; and nearly 20% have four or all five risk factors. This study also shows that when considering single behavioural risk factors in isolation, one would reasonably expect that a substantial proportion of the population will not have the risk factor in question. However, even the most prevalent risk factor - poor diet - is present in 80% of the population. But only 2.5% of the population was without any of the five behavioural risk factors. Is this surprising? Our analysis shows that around two-thirds of the Scottish population is overweight or obese, a similar proportion are not sufficiently physically active, and most people have a poor diet - it is just that it is not the same majority for each factor. The most important determinants of multiple risk factors were low educational attainment and residence in the most deprived communities.

The main limitation of our study is common to most studies investigating prevalence of risk factors in a population - that is, it is a cross-sectional survey and therefore cannot be used to determine causal associations. Furthermore, the detailed pathways and mechanisms between the socioeconomic determinants and the risk factors investigated cannot be fully determined from this study.

These behaviours were self-reported and are not all externally validated or entirely objective measures. Respondents might tend to give answers that would convey more favourable behaviours. This was confirmed for alcohol consumption by an analysis comparing self-reported alcohol intake in the Scottish Health Surveys with alcohol sales estimates which suggested that surveys may understate alcohol consumption by as much as 50% [10]. Validation of self-reported smoking data using salivary cotinine levels found that that the proportion of men who smoked rose from 32% (self-reported) to 35% (validated), and the proportion of women from 28% to 31%, indicating some under-reporting of smoking [12]. Nevertheless, the Scottish Health Survey is recognised as providing a useful source of data to quantify behaviours and health at the population level with no evidence of substantial socioeconomic response bias [12,19].

For most of the risk factors, presence or absence is relatively straightforward. Diet and nutrition however is a much more complex behaviour than other risk factors. In order to simplify it to a binary dietary measure fruit and vegetable consumption in line with current recommendations was used. This is only one aspect of diet and nutrition, and in terms of healthy weight, does not necessarily mean that total calorie consumption is within certain limits for example. Overall, therefore, the most important aspects of diet are likely to be: total calorie consumption; total fat consumption; salt consumption; sugar consumption; and fruit and vegetable consumption. Therefore, ideally a dietary risk factor should consider each of these. Nevertheless, for the purposes of comparison with other studies and as a measure of diet recognised to be particularly important to health we utilised a variable related to fruit and vegetable consumption. It is increasingly recognised that overweight and obesity are being investigated as separate categories [20], and analysis in this way is likely to have highlighted further the associations between deprivation and obesity.

The socioeconomic measures used in this analysis are not necessarily entirely representative of all aspects of socioeconomic circumstances. Area-based deprivation, individual-level educational attainment, marital status, occupational social class, employment activity status and ethnicity do not capture the full picture of social, economic, and demographic determinants. For example, individual and household income are known to be related to risk factor behaviours but were not available in this analysis. Residual confounding by socioeconomic status therefore remains a possibility.

The strengths of the analysis include the rigour of the methods used for the Scottish Health Survey. The response rate for the survey was 67% of all eligible households. The large population sample is reflected in the precision and tight confidence interval of prevalence estimates. However, it is likely that a lower percentage of individuals would have participated which potentially could reduce the representative of the response and increase the risk of socioeconomic bias. The Scottish Health Survey is generally considered to be a socioeconomically representative sample [12,19], however the full data available in this analysis indicate some skewing of response to those less deprived quintiles. Therefore any participation bias would likely contribute to even greater associations with low socioeconomic status/circumstances. We also found no differences in the SIMD profile of those included with all five risk factors compared with those excluded. The survey used weights to correct for survey design (large households were underrepresented) and non-response biases [12]. Furthermore, the age distribution corresponds to the 2003 General Register Office for Scotland (GROS) mid-year population estimates, where the proportion of the adult population in the age-groups (used in this analysis) were: 16-24 years - 14.2%; 25-34 years - 15.7%; 35-44 years - 19.3%; 45-54 years - 16.6%; 55-64 years - 14.3%; and 65+years - 19.9%.

Comparing our findings with analyses of combinations of multiple risk factors from health surveys from across the world (Table 4), the Scottish population seems to have among the lowest rates of absence of any behavioural risk factors and highest rates of multiple risk factors. Higher prevalence of multiple risk factors was observed in Scotland than in USA [5,8], Finland [6], Netherlands [7], Switzerland [9], New Zealand [21], and Canada [22], although similar findings were observed for the English population in the same year [23]. The strong associations of multiple risk factors with low socioeconomic status observed in the Scottish population were found noted in other surveys - particularly low educational attainment [5-8], but also low occupational social class [23]. This study uniquely found strong associations with both area-based and individual level socioeconomic measures and clustering of risk factors.

**Table 4:** Comparison with health surveys from across the world

Reference	Setting/Country	Study Design	Risk Factors Included in Study	Main Findings	Comparing risk factor combinations in common with our data
Poortinga (2007) [23]	England 2003	11,492 subjects in the Health Survey for England	smoking risky alcohol drinking physical inactivity diet low in fruit/vegetables	26% had 3+ risk factors 6% had no risk factors	27% had 3+ risk factors 6% had no risk factors

Tobius et al (2007) [21]	New Zealand 2002/03	Over 17,000 subjects in New Zealand Health Survey	smoking risky alcohol drinking physical inactivity diet low in fruit/vegetables	13% had 3+ risk factors 29% had no risk factors.	27% had 3+ risk factors 6% had no risk factors

Chiolero et al (2006) [9]	Switzerland 2002	18,000 subjects from the 2002 Swiss Health Survey	smoking risky alcohol drinking physical inactivity diet low in fruit/vegetables	8% had 3+ risk factors 30% had no risk factors	27% had 3+ risk factors 6% had no risk factors

Fine et al (2004) [8]	USA 2001	30,000 subjects from the National Health Interview Survey	smoking risky alcohol drinking physical inactivity overweight	17% had 3+ risk factors 10% had no risk factors	41% had 3+ risk factors 7% had no risk factors

Klein-Geltink et al (2006) [22]	Canada 2000	Over 100,000 subjects in the Community Health Survey	smoking risky alcohol drinking physical inactivity overweight	8% had 3+ risk factors 21% had no risk factors	41% had 3+ risk factors 7% had no risk factors

Schuit et al (2002) [7]	Netherlands 1993-97	17,000 subjects in the Dutch population survey	smoking risky alcohol drinking physical inactivity diet low in fruit/vegetables	20% had 3+ risk factors 10% had no risk factors	27% had 3+ risk factors 6% had no risk factors

Laaksonen et al (2001) [6]	Finland 1991-98	23,000 subjects in the Health Behaviour Among Finnish Adult Population project	smoking risky alcohol drinking physical inactivity diet low in fruit/vegetables	10% had 3+ risk factors 33% had no risk factors	27% had 3+ risk factors 6% had no risk factors

Berrigan et al (2003) [5]	USA 1988-94	15,000 subjects from the Third National Health and Nutrition Survey	smoking risky alcohol drinking physical inactivity diet low in fruit/vegetables diet high in fat.	18% had 3+ risk factors 6% had no risk factors	27% had 3+ risk factors 6% had no risk factors

The health implications for individuals of multiple behavioural risk factors are underexplored. There are few examples where combined behavioural risk factors are implicated in aetiology. Combined smoking and alcohol are reported to synergistically increase the risk of upper aerodigestive tract cancer [24]; and combinations of the "Framingham" risk factors which include smoking, and physical inactivity have been found to account for most of the risk associated with cardiovascular disease [25].

Socioeconomic determinants seem to have an effect on multiple behavioural risk factors at both the individual and area-level. Both low educational attainment and residence in a deprived community were strongly associated with multiple risk factors.

The role of educational attainment in health and in health behaviours is yet to be fully 'unbundled' [26]. Potential mechanisms could include low education level: (i) acting as a direct causal effect - as it is generally fixed in early life it may also reflect childhood experiences [27]; (ii) influencing position in society and the inferred stresses [28,29]; (iii) reflecting income and access to health care and health information [30]; (iv) influencing occupation [31]; (v) determining values for the future [32]; (vi) as a means of developing cognitive skills and so decision-making [32]; (vii) affecting preferences and so locus of control [32]; and (viii) determining social/peer networks [32].

The explanation of the effect of residence in an area of high deprivation on the prevalence of multiple risk factors is worth considering. Deprivation is measured here by SIMD - an area-based socioeconomic measure deprivation. While use of area measures has previously been criticised as producing an 'ecological fallacy' - as individuals are allocated an area socioeconomic status based on their residence - this may in fact help with an explanation. A convincing case that the ecological perspective (and the way it is measured in terms of socioeconomic level) can provide important insights has been proposed [33,34]. The argument follows that the socioeconomic environment affects health and wellbeing apart from or over and above that of the individual. Macintyre and Ellaway's (2000) distinction between contextual (place related) and compositional (people related) are the key elements in this multi-level perspective [34].

Area deprivation could impact on behavioural risk factors through a range of potential pathways, including: (i) economic and social deprivation related to the physical environment (e.g. healthy food access, availability of low cost alcohol, poor housing, environmental pollution, transport, recreational facilities); (ii) economic and social deprivation related to the social environment (including 'social trauma' from e.g. fear of crime, social isolation, discrimination; and 'physical trauma' from e.g. alcohol, smoking culture); (iii) targeted marketing of harmful products to deprived area; (iv) inadequate area-services (e.g. education, health, transport, recreation).

A potential explanation for the relationship between socioeconomic determinants and multiple risk factors is "cultural". Frolich et al (2001) describe the 'collective lifestyles' model of community behaviour as potentially a way of capturing the collective or cultural dimension of behaviours [35]. They described this as behaviours being integral to social practices and norms. Continuing the cultural explanation, Hanlon et al. (2005) recently explored the possibility of what they described as a 'Scottish effect' to explain higher mortality rates in Scotland than in England and Wales between 1981 and 2001, when a decreasing influence of socioeconomic deprivation was observed in the data. While the 'Scottish effect' was not fully defined, one interesting possibility raised was the cultural explanation. This was described as arising from social factors and in particular deprivation, which potentially impact on the collective psyche, affecting health through behaviours [36].

Whereas health and social services input to addressing risk factors have generally focussed on individual risk factors, the move towards 'anticipatory care' in Scotland is leading to a more holistic approach exemplified by the national anticipatory care programme 'Keep Well' [37]. This recognises that there may be more than one risk factor present in individuals. Assessment through anticipatory care can lead to a plan of action for an individual taking into account readiness to change, and considering the other risk factors and the socioeconomic context.

On a population-wide basis we need to further improve aspects of the physical and social and economic environment which predispose to alcohol misuse, smoking, lack of physical activity and poor nutrition. Recent policy documents from the Scottish Government show a level of commitment to legislation in relation to smoking and alcohol. There is also a commitment to looking at healthy weight, although policy in relation to the economy and in particular industries in food, energy and transport seems to override this. There is a real need to bring these policies together with social and economic policy to ensure change. The socioeconomic determinants of these behavioural risk factors need to be more explicitly acknowledged and understood.

## Conclusions

It is reasonable to conclude that the vast majority of the population have something to gain in terms of current or future health by identifying and addressing risk factors. Healthy behaviours do not seem to cluster while unhealthy behaviours cluster - this is important insight into how to tackle risk factors both from a population public health and individual patient perspective. Furthermore these risk factors are strongly associated with low socioeconomic circumstances. Health services, health improvement, and anticipatory care approaches and policy need to respond by becoming more joined up. These findings also support the continuation in efforts to tackle health inequalities via both a population and individual high-risk approaches to prevention and risk reduction.

## Abbreviations

BME: Black and Minority Ethnic groups; BMI: Body Mass Index; SIMD: Scottish Index of Multiple Deprivation

## Competing interests

The authors declare that they have no competing interests.

## Authors' contributions

All authors developed the study design. DIC, OH, RL planned and coordinated the work. RL undertook the statistical analysis. DIC wrote the first draft of the manuscript and coordinated feedback. All authors contributed to revising manuscript, and all authors read and approved the final manuscript.

## Pre-publication history

The pre-publication history for this paper can be accessed here:

http://www.biomedcentral.com/1471-2458/10/330/prepub
